# Enhancing exposure therapy for posttraumatic stress disorder (PTSD): a randomized clinical trial of virtual reality and imaginal exposure with a cognitive enhancer

**DOI:** 10.1038/s41398-022-02066-x

**Published:** 2022-07-27

**Authors:** JoAnn Difede, Barbara O. Rothbaum, Albert A. Rizzo, Katarzyna Wyka, Lisa Spielman, Christopher Reist, Michael J. Roy, Tanja Jovanovic, Seth D. Norrholm, Judith Cukor, Megan Olden, Charles E. Glatt, Francis S. Lee

**Affiliations:** 1 Department of Psychiatry, Weill Cornell Medical College, New York, NY USA; 2 Emory University School of Medicine, Atlanta, GA USA; 3 University of Southern California Institute for Creative Technologies, Los Angeles, CA USA; 4 Department of Rehabilitation and Human Performance, Icahn School of Medicine at Mount Sinai, New York, NY USA; 5 Department of Psychiatry, VA Long Beach Healthcare System, Long Beach, CA USA; 6 University of California, Irvine, Irvine, CA USA; 7 Science 37, Los Angeles, CA USA; 8 Department of Medicine and Center for Neuroscience and Regenerative Medicine, Uniformed Services University of the Health Sciences, Bethesda, MD USA; 9 Department of Psychiatry and Behavioral Neurosciences, Wayne State University, Detroit, MI USA

**Keywords:** Psychology, Genetics

## Abstract

Posttraumatic stress disorder (PTSD) is a significant public health issue. Yet, there are limited treatment options and no data to suggest which treatment will work for whom. We tested the efficacy of virtual reality exposure (VRE) or prolonged imaginal exposure (PE), augmented with D-cycloserine (DCS) for combat-related PTSD. As an exploratory aim, we examined whether brain-derived neurotrophic factor (BDNF) and fatty acid amide hydrolase (FAAH) moderated treatment response. Military personnel with PTSD (*n* = 192) were recruited into a multisite double-blind randomized controlled trial to receive nine weeks of VRE or PE, with DCS or placebo. Primary outcome was the improvement in symptom severity. Randomization was stratified by comorbid depression (MDD) and site. Participants in both VRE and PE showed similar meaningful clinical improvement with no difference between the treatment groups. A significant interaction (*p* = 0.45) suggested VRE was more effective for depressed participants (CAPS difference M = 3.51 [95% CI 1.17–5.86], *p* = 0.004, ES = 0.14) while PE was more effective for nondepressed participants (M = −8.87 [95% CI −11.33 to −6.40], *p* < 0.001, ES = −0.44). The main effect of DCS vs. placebo was not significant. Augmentation by MDD interaction (*p* = 0.073) suggested that depressed participants improved more on placebo (M = −8.43 [95% CI −10.98 to −5.88], *p* < 0.001, ES = −0.42); DCS and placebo were equally effective for nondepressed participants. There was an apparent moderating effect of BDNF Val66Met polymorphism on DCS augmentation (ES = 0.67). Met66 allele carriers improved more on DCS (ES = −0.25). FAAH 385 A carriers improved more than non-carriers (ES = 0.33), particularly those with MDD (ES = 0.62). This study provides a step toward precision therapeutics for PTSD by demonstrating that comorbid MDD and genetic markers may help guide treatment selection.

ClinicalTrials.gov Identifier: NCT01352637.

## Introduction

PTSD remains a significant worldwide public health problem a generation after the World Trade Center attacks of September 11, 2001, which played a substantial role in underscoring the paucity of effective treatments for PTSD. PTSD is common following a wide variety of traumas, including combat exposure, vehicle accidents, sexual assault, and interpersonal violence. An estimated 3.9% of the adult population worldwide is at risk for PTSD during their lifetime [1]. PTSD is associated with multiple adverse outcomes, including suicide [2], psychiatric co-morbidity, marital discord, medical illness, unemployment, absenteeism, and an estimated annual PTSD-related productivity loss of approximately $3 billion for the U.S. economy [2]; without treatment PTSD becomes chronic [3]. All recent expert consensus guidelines recommend exposure therapy as a first-line treatment for PTSD. Though it is not effective for everyone [4], many patients no longer meet the criteria for PTSD following treatment and symptom reduction in treatment-responders is typically maintained long-term [5]. A recent systematic review identified comorbid depression as a significant predictor of worse outcomes in those with PTSD, which highlights the need for further exploration of what therapies are most effective in the face of comorbid depression [6] and consensus on intervention strategies.

While pharmacologic treatments are not currently recommended as stand-alone first-line treatments for PTSD [7], novel pharmacotherapeutics that augment existing therapies offer an alternative. D-cycloserine (DCS) is a partial agonist at the N-methyl-D-aspartate (NMDA) glutamate receptor, which plays an essential role in mediating learning and memory. Specifically, fear extinction is blocked by NMDA receptor antagonists in animal models [8]. Preclinical research has demonstrated that DCS facilitates extinction learning and memory in rodents [9], which provides the rationale for several clinical studies suggesting that DCS can enhance exposure-based treatment for PTSD [10–12]. However, the results of PTSD treatment studies have been mixed [11].

No biomarkers have yet been proven to predict treatment response in PTSD. There is speculation that BDNF may help explain individual differences in treatment response. BDNF gene is important for neural plasticity and human memory [13]. Research using a knock-in mouse model containing the human variant Val66Met (rs6265) in the gene coding for brain-derived neurotrophic factor (BDNF), as well studies in human carriers with this BDNF variant has shown that both mice and human carriers exhibited impaired fear extinction [14], which may be a specific biomarker of PTSD treatment response [15]. Moreover, BDNF is important for neural plasticity and human memory leading some researchers to speculate that BDNF may help explain individual differences in trauma treatment outcomes. Similarly, recent research on the human genetic variant C385A (rs324420), in the endocannabinoid modifying enzyme, fatty acid amide hydrolase (FAAH), in both knock-in mice and human carriers suggest that it enhances fear extinction learning [16] and may therefore moderate response to exposure therapy also.

Our objective was to determine relative efficacy of VRE and PE, each in combination with DCS or placebo. The co-primary hypotheses were 1) DCS will augment response to exposure therapy (VRE or PE) for PTSD; 2) VRE will be more effective than PE. The secondary hypotheses were 1) there will be an interaction between DCS and mode of exposure therapy such that DCS + VRE will reduce PTSD symptom severity more over the first 5 sessions than other treatment combinations, and 2) DCS augmentation of exposure therapy will be greater for participants with the BDNF Met66 allele. Randomization was stratified by comorbid depression. Exploratory analyses tested whether there was a differential response to the treatments among those with and without MDD. An exploratory aim was to assess BDNF Val66Met and FAAH C385A as genetic moderators of treatment response. Additionally, we proposed to evaluate additional promising genetic markers as they become available during this study. FAAH was selected because recent animal and human studies suggest it plays a role in fear extinction learning and hence has a potential utility in determining for whom exposure-based therapies will be most efficacious. Thus, we evaluated whether the response to exposure therapy was greater for subjects with the FAAH C385A polymorphism, across both treatments and by comorbid depression.

## Methods

### Study design, randomization and masking

Detailed study procedures are available in Difede et al. [17]. This was a 2×2 (DCS vs. placebo and VRE vs. PE) multisite randomized double-blind treatment study for post-9/11 combat-related PTSD. Participants received 9 sessions of treatment. Randomization was stratified by comorbid depression (MDD) and site. Upon eligibility, participants were randomized to VRE or PE by the study coordinators according to allocation lists prepared by the study statistician prior to the study start. Site pharmacies performed randomization to the medication condition. Independent assessors, blinded to therapy and medication conditions, assessed PTSD and other psychopathology at baseline, mid-treatment, post-treatment, and 3-month follow-up. All study personnel and participants were blinded to medication condition. The study was approved by each site’s Institutional Review Board, the Office of Human Research Protection, U.S. Army Medical Research and Materiel Command (USAMRMC) and monitored by a data and safety monitoring board. Written consent was obtained from each potential participant. All participants were informed regarding the study purpose and risks.

### Participants

Participants were U.S. military service members of any duty status and veterans who served in Operations Iraqi Freedom and Enduring Freedom, or other later operations in Iraq or Afghanistan (e.g., Operation New Dawn). Participants were seen from April 1, 2011 through April 5, 2018 at three diverse geographic sites: a civilian site (Weill Cornell Medical College in New York City), a VA site (Veterans Administration Long Beach Healthcare System, Long Beach, California) and an active duty site (Walter Reed National Military Medical Center, Bethesda, Maryland). Inclusion criteria were: diagnosis of OEF-OIF (Operations Enduring Freedom or Iraqi Freedom, or other later operations) combat-related PTSD; female participants of childbearing potential must agree to use an effective method of birth control (i.e., oral contraceptive, Norplant, diaphragm, condom, or spermicide) during the course of the study, or to remain abstinent from sex, to ensure they do not become pregnant during the course of the study; ability to provide informed consent and function at an intellectual level sufficient to allow accurate completion of all assessment instruments; participants must be literate in English; participants must be medically health and willing to take the study medication; participant’s trauma must be consistent with available VRE stimuli. Exclusion criteria were: lifetime or current diagnosis of schizophrenia or other psychotic disorder, bipolar disorder; participation in a clinical trial during the previous 3 months; current evidence or history of significant unstable medical illness or organic brain impairment, including stroke, CNS tumor, demyelinating disease, cardiac, pulmonary, gastrointestinal, renal, or hepatic impairment; participants who in the investigator’s judgment pose a current suicidal or homicidal risk; alcohol, medical, or substance dependence within the past 90 days other than nicotine or caffeine; treatment with any other concomitant medication with primarily CNS activity or treatment with any medication that the PI judges not acceptable for this study; history of seizures; pregnancy or lactation.

### Procedures

#### Treatment [17]

Exposure therapy was delivered in nine 90-minute individual weekly sessions. Both manualized study interventions followed guidelines for exposure therapy for PTSD [18] and were identical in timing and structure except for the mode of exposure. In the first two sessions, therapists gathered information and provided an explanation of PTSD, common reactions to trauma, treatment rationale, and taught a breathing retraining technique. The remaining seven sessions consisted of a brief check-in, 30–45 minutes of exposure and 30 minutes of processing discussion. No homework was assigned to ensure all exposures occurred under the study drug experimental conditions.

Prolonged Exposure (PE). The imaginal exposure element of PE followed standard instructions. Briefly, the participant was instructed to close their eyes, imagine the scene of their trauma, and repeatedly recount the trauma vividly, aloud, and in the present tense.

Virtual Reality Exposure (VRE). The VR-enhanced exposure followed the same instructions, except that participants wore VR equipment and were exposed to virtual simulations of common combat scenarios while recounting their trauma [19]. Participants wore a Head Mounted Display (HMD) with integrated head-tracking and stereo earphones. Participants moved in the virtual environment using a handheld controller. The therapist controlled the environment and stimuli on a separate computer, and simulated the trauma memory as the patient recounted it. Virtual environments included Humvee/convoy scenarios and numerous patrol environments in urban, rural, mountain, and desert settings, among others. Stimulus options included sounds of weapon fire, explosions, incoming mortars, helicopter flyovers, vehicle noise, wind, human voices, and radio; visual stimuli included night vision, wounded civilians and combatants, and wrecked vehicles. Tactile stimuli (i.e., vibration) were delivered through a raised platform with an attached subwoofer. The therapist communicated with the participant via a microphone and earphones. The participant removed the HMD prior to processing.

All therapists provided both types of therapy (VRE and PE), in the order determined by the randomization scheme. Therapists received individual supervision for each session during training and for their first VRE and PE patient. Thereafter, supervision was provided weekly in group conference calls. Eighteen percent of therapy sessions were randomly selected for treatment protocol adherence ratings conducted by independent clinical experts in the treatments (VRE *n* = 108 and PE = 66). Adherence was high, with more than 99% of essential elements observed during both the VRE and PE sessions. There were no observed incidents of clinicians implementing interventions that were not in the treatment manual.

Study drug. Beginning at session 3 (the first exposure), participants were administered a pill upon arrival. The session began 30 minutes afterwards.

#### Outcomes

The primary outcome was the Clinician Administered PTSD Scale (CAPS-IV) [20] severity score (range 0–136). MDD and other psychiatric diagnoses were assessed using the Mini International Neuropsychiatric Interview (MINI) [21], a structured diagnostic interview for DSM-IV. Twenty percent of assessments were randomly selected for interrater reliability (CAPS, *n* = 144; MINI depression module, *n* = 80). The intraclass correlation for CAPS-IV severity was 0.98 and 0.99 for the past month and past week, respectively. The level of agreement for MDD diagnosis (presence/absence) was also high (κ = 0.95).

Secondary outcomes were self-reported posttraumatic stress symptoms on the Posttraumatic Stress Disorder Checklist [22] and depressive symptoms on the Beck Depression Inventory [23].

Lifetime trauma history was assessed using the Trauma History Questionnaire [24]. Participant treatment preference (VRE vs. PE) was assessed using a form developed for this study. Participant satisfaction and expectancy were assessed using Client Satisfaction and Client Expectancy Questionnaires [25].

Saliva samples for genotyping were collected using the Oragene system (DNA Genotek) and assayed for BDNF Val66Met (rs6265) and FAAH C385A (rs324420). Assays were conducted using Taqman assays (ABI: rs6265, C__11592758_10; rs324420, C___1897306_10).

### Statistical analysis

Sample size was planned to provide adequate power (≥0.80, at multiplicity-adjusted two-tailed alpha = 0.025) to detect an effect size of 0.30, which corresponds to a difference of 9 units on the CAPS (*SD* = 30).

The co-primary hypotheses were tested using separate mixed-effects multivariable linear models predicting change in CAPS scores (past week). Models included fixed effects for time (baseline, after sessions 4 and 6, and posttreatment), exposure therapy (VRE vs. PE) or augmentation (DCS vs. placebo), and their interaction with time. The models also included fixed effects for site and baseline MDD; their interactions with exposure therapy or augmentation were retained if significant at the pre-specified alpha=0.10. First-order autoregressive models with random intercepts and slopes were tested against unstructured covariance models to determine best model fit using likelihood ratio tests; in every case the latter models were superior. Models were adjusted for covariates significantly associated with baseline CAPS score (prior PTSD treatment *r* = 0.14, *p* = 0.046; concussion *r* = 0.19, *p* = 0.007, history of physical or sexual trauma *r* = 0.19, *p* = 0.008).

Fixed effects were evaluated using F-tests within the model. Post hoc comparisons within significant interaction effects were conducted using univariate t-tests on model-estimated means. Effect sizes (ES) were computed by dividing model estimated between-group differences at post-treatment by the common standard deviation of the change scores baseline-posttreatment.

The primary analysis was of the intent-to-treat sample. Secondary analyses compared treatment completers only. All analyses were performed using SPSS version 25 (Armonk, NY).

Interpretation of the exploratory genetic hypotheses followed Kraemer et al. [26], who recommends comparing the magnitude of baseline-posttreatment standardized change scores. A moderating effect was considered present if a substantial difference in effect size was observed between genotype groups. The first analysis compared CAPS-IV change scores between the augmentation group (DCS vs. placebo) for participants with and without the BDNF Met allele. The second analysis compared CAPS-IV change scores across treatment groups for participants with and without FAAH C385A allele. Additional FAAH C385A analysis was stratified by baseline MDD, as this was significant in the primary analyses.

## Results

### Participant flow

**Fig. 1:**
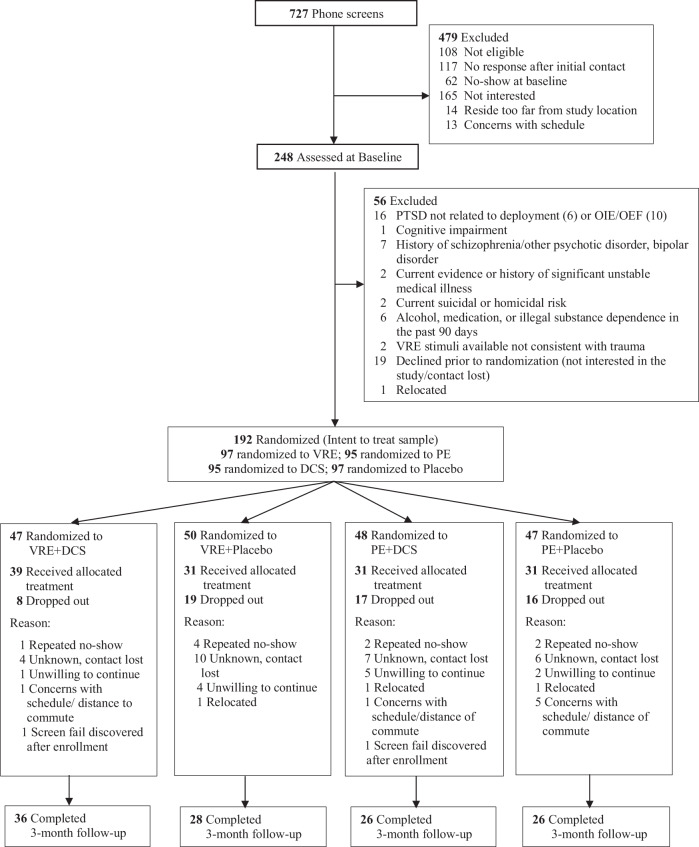
Consort flow diagram. *Note*: Detailed information on patient enrollment throughout the study. VRE virtual reality exposure therapy, PE prolonged imaginal exposure therapy, DCS D-cycloserine, OEF and OIF operations Iraqi freedom and enduring freedom.

This multi-site double-blind randomized controlled trial recruited U.S. military personnel diagnosed with PTSD between April 1, 2011, and April 5, 2018, at 3 sites: one civilian (which accepted military personnel of any duty status), one VA, and one active-duty site. Of the 727 screened individuals, 248 completed baseline assessments, 192 were randomized, and 132 (68.8%) participants completed treatment (VRE *n* = 70 (72.2%), PE *n* = 62 (65.3%); DCS *n* = 70 (73.7%), placebo *n* = 62 (63.9%) (Fig. 1).

### Demographic, military service and clinical characteristics

Participants were mostly men (*n* = 172, 90%), White (*n* = 88, 45.8%), married or living with significant other (*n* = 100, 52.1%) with at least some college education (*n* = 163, 84.9%). The mean age was 34.62 (SD = 7.80, range 21–58). The majority had one (*n* = 68, 35.4%) or two deployments (*n* = 66, 34.4%). Twenty-four percent (*n* = 46) were active duty at baseline assessment.

The mean CAPS-IV severity at baseline was 73.04 (SD = 19.47, range 28–130), with the majority in the extreme or severe range (*n* = 171, 89.1%). Half (*n* = 104, 54.2%) had co-morbid MDD and 23.4% (*n* = 45) were on a stable dose of selective serotonin reuptake inhibitors (SSRIs). Participant characteristics were overall balanced among groups (Table 1).

**Table 1:** Baseline Demographic, Military Service and Clinical Characteristics.

	All *N* = 192		VRE *n* = 97		PE *n* = 95		*p* value	DCS *n* = 95		Placebo *n* = 97		*p* value
**Demographic characteristics**												
Age M (SD)	34.62	7.80	34.29	7.32	34.96	8.30	0.556	34.97	8.17	34.27	7.45	0.538
Male n (%)	172	89.6	88	90.7	84	88.4	0.602	83	87.4	89	91.8	
Ethnicity/Race n (%)							0.975					0.676
White	88	45.8	46	47.4	42	44.2		41	43.2	47	48.5	
African American/Black	29	15.1	14	14.4	15	15.8		14	14.7	15	15.5	
Hispanic/Latino	53	27.6	26	26.8	27	28.4		30	31.6	23	23.7	
Other	22	11.5	11	11.3	11	11.6		10	10.5	12	12.4	
Education n (%)							0.479					0.685
High school or GED	29	15.1	17	17.5	12	12.6		12	12.6	17	17.5	
Some college/training	104	54.2	54	55.7	50	52.6		52	54.7	52	53.6	
College graduate	33	17.2	13	13.4	20	21.1		16	16.8	17	17.5	
More than college	26	13.5	13	13.4	13	13.7		15	15.8	11	11.3	
Relationship status n (%)							0.915					0.749
Single	59	30.7	30	30.9	29	30.5		27	28.4	32	33.3	
Married/Live w/significant other	100	52.1	49	51.0	51	53.7		52	54.7	48	50.0	
Separated/Divorced/Widowed	33	17.2	17	18.5	15	15.8		16	16.8	16	16.7	
**Military characteristics**												
Military service n (%)							0.119					0.492
OEF only	39	20.3	25	64.1	14	35.9		16	41.0	23	59.0	
OIF only	86	44.8	38	44.2	48	55.8		44	51.2	42	48.8	
Both OEF and OIF	67	34.9	34	50.7	33	49.3		35	52.2	32	47.8	
Branch of the Armed Forces n (%)							0.357					0.787
Army	113	58.9	62	54.9	51	45.1		56	49.6	57	50.4	
Marines	55	28.6	26	47.3	29	52.7		26	47.3	29	52.7	
Navy	15	7.8	5	33.3	10	66.7		8	53.3	7	46.7	
Air Force	8	4.2	3	37.5	5	62.5		5	62.5	3	37.5	
Other	1	0.5	1	100	0	0		0	0	1	100	
Number of deployments n (%)							0.198					0.576
1	68	35.4	38	55.9	30	44.1		35	51.5	33	48.5	
2	66	34.4	33	50.0	33	50		32	48.5	34	51.5	
3	26	13.5	15	57.7	11	42.3		10	38.5	16	61.5	
4 or more	32	16.7	11	34.4	21	65.6		18	56.3	14	43.8	
Months in theater M (SD)	19.19	13.48	17.79	10.57	20.62	15.85	0.149	19.31	14.94	19.08	11.97	0.909
Active duty n (%)	46	24	22	47.8	24	52.2	0.675	23	50.0	23	50.0	0.935
**Clinical characteristics**												
Past PTSD treatment n (%)	118	61.5	58	49.2	60	50.8	0.632	57	48.3	61	51.7	0.681
Traumatic Brain Injury n (%)	65	34.6	34	35.0	31	32.9	0.804	32	33.7	33	34.0	0.953
Concussion n (%)	57	29.7	25	25.8	32	33.7	0.230	28	29.5	29	29.9	0.949
Physical or sexual trauma n (%)	124	65.3	65	67.0	59	63.4	0.605	59	62.8	65	67.7	0.474
Baseline MDD n (%)	104	54.2	54	51.9	50	48.1	0.673	52	50.0	52	50.0	0.875
Baseline SSRI n (%)	45	23.4	16	16.5	29	30.5	0.022	23	24.2	22	22.7	0.802
Alcohol Abuse n (%)	46	24.0	25	25.8	21	22.1	0.552	20	21.1	26	26.8	0.351
Substance Abuse n (%)	12	6.3	5	5.2	7	7.4	0.526	6	6.3	6	6.2	0.970

*P* values are based on independent samples *t*-tests or chi-squared tests (*p* < 0.05 are bolded). Alcohol and substance abuse include participants meeting MINI criteria for abuse (past 12 months) and dependence (past 12 months but not past 3 months). Lifetime Traumatic Brain Injury was self-reported. Concussion information was self-reported as head trauma involving loss of consciousness for more than 5 minutes. TBI was Abbreviations: *VRE* virtual reality exposure therapy, *PE* prolonged imaginal exposure therapy, *DCS* D-Cycloserine, *MDD* major depressive disorder, *SSRI* Selective serotonin reuptake inhibitor, *CAPS-IV* Clinician Administered Posttraumatic Stress Disorder (PTSD) Scale for DSM-IV, *OEF* and *OIF* Operations Iraqi Freedom and Enduring Freedom.

### Treatment preference, treatment received and treatment satisfaction

Most participants expressed preference for VRE treatment (*n* = 145, 76.7%) (Fig. S1). Neither treatment preference nor treatment satisfaction were associated with treatment outcome (Table S1).

### Treatment dropout and adverse events

The dropout rate was 31.3% (*n* = 60). However, more than half of dropouts occurred prior to exposure: 13 (22%) dropped prior to session 1, another 19 (32%) dropped before the first exposure during session 3. Only 8 participants dropped between the first and second exposure sessions. Of note, drop out, while not statistically significant, was lowest in VRE and DCS (VRE 27.8% (*n* = 27), PE 34.7% (*n* = 33), *p* = 0.302; DCS 26.3% (*n* = 25), placebo 36.1% (*n* = 35), *p* = 0.144). Dropout was particularly low in VRE + DCS condition (*n* = 8, 17%). Dropout was associated with physical/sexual abuse history (Table S2). Dropout among those with an abuse history was 32.3% in VRE and 44.1% in PE. No adverse events were reported.

### Primary outcome

#### Exposure Therapy (VRE vs. PE)

**Fig. 2:**
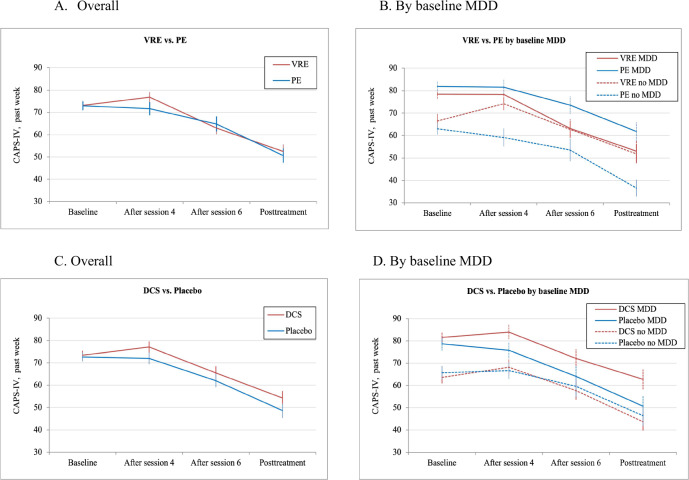
Cross-sectional mean CAPS-IV (past week) scores by group over time. *Note*: Cross-sectional mean CAPS-IV (past week) scores: exposure therapy over time overall (**A**) and by baseline MDD (**B**); augmentation over time overall (**C**) and by baseline MDD (**D**). Bars represent standard errors. VRE virtual reality exposure therapy, PE prolonged imaginal exposure therapy, DCS D-cycloserine, MDD major depressive disorder.

There was a significant effect of time (F = 51.18, *p* < 0.001), but neither the main effect for therapy type (F = 2.36, *p* = 0.126), nor the therapy-by-time interaction (F = 0.295, *p* = 0.587) were significant. Symptom improvements were 19.98 points in VRE and 21.23 points in PE (model-estimated CAPS mean difference at posttreatment M = 0.01 [95% CI −3.86 to 3.87]. A significant effect of baseline MDD (F = 26.88, *p* < 0.001) indicated that nondepressed participants had lower symptoms during treatment. A significant therapy-by-MDD interaction (F = 4.07, *p* = 0.045) suggested that VRE was more effective for depressed participants (CAPS mean difference at posttreatment M = 3.51 [95% CI 1.17 to 5.86], *p* = 0.004, ES = 0.14) but PE was more effective for nondepressed participants (CAPS mean symptom difference at posttreatment M = −8.87 [95% CI −11.33 to −6.40], *p* < 0.001, ES = −0.44) (Table 2, Fig. 2).

**Table 2:** Descriptive and Model-estimated Statistics for Primary Outcome (CAPS-IV, past week): Exposure Therapy and Augmentation Over Time by Baseline MDD.

	Exposure Therapy (VRE vs. PE)						Augmentation (DCS vs. Placebo)					
	VRE *n* = 97			PE *n* = 95			DCS *n* = 95			Placebo *n* = 97		
CAPS-IV, past week	Descriptive Statistics						Descriptive Statistics					
Overall	N	M	SD	N	M	SD	N	M	SD	N	M	SD
Baseline	97	73.13	19.48	95	72.94	19.56	95	73.42	19.31	97	72.66	19.72
After session 4	81	76.85	19.88	68	71.65	24.48	73	77.04	21.39	76	72.01	22.77
After session 6	73	62.97	23.73	64	64.80	26.78	70	65.51	25.31	67	62.06	24.99
Posttreatment	69	52.42	26.55	61	50.61	25.22	69	54.20	26.72	61	48.59	24.71
3-month Follow-up	64	49.03	26.12	52	50.35	28.93	62	53.00	27.55	54	45.74	26.71
**MDD**												
Baseline	54	78.44	17.20	50	81.90	17.28	52	81.54	16.86	52	78.67	17.66
After session 4	45	78.31	22.23	38	81.55	21.81	41	83.93	20.41	42	75.76	22.91
After session 6	39	63.15	25.62	36	73.53	23.77	38	72.13	25.45	37	64.03	24.46
Posttreatment	35	53.00	29.02	34	61.76	24.00	38	62.74	27.34	31	50.68	25.06
3-month Follow-up	30	53.10	29.45	27	61.67	29.84	31	64.00	28.49	26	49.00	29.54
**No MDD**												
Baseline	43	66.47	20.31	45	62.98	17.10	43	63.60	17.58	45	65.71	19.87
After session 4	37	74.14	17.26	30	59.10	22.06	32	68.22	19.55	35	66.66	22.15
After session 6	34	62.76	21.74	28	53.57	26.61	32	57.66	23.14	30	59.63	25.83
Posttreatment	34	51.82	24.17	27	36.56	19.20	31	43.74	22.16	30	46.43	24.59
3-month Follow-up	32	46.22	23.08	22	39.86	23.55	29	43.17	22.19	25	44.16	24.91
**CAPS-IV, past week**	**Model-estimated statistics**^**a**^ **at posttreatment**						**Model-estimated statistics**^**a**^ **at posttreatment**					
	Mean difference (VRE vs. PE)		95%CI		*p* value	ES	Mean difference (DCS vs. placebo)		95%CI		*p* value	ES
MDD												
Intent-to-treat analysis	3.51		(1.17, 5.86)		0.004	0.14	−8.43		(−10.98, −5.88)		<0.001	−0.42
No MDD												
Intent-to-treat analysis	−8.87		(−11.33, −6.40)		<.001	−0.44	0.75		(−1.81, 3.30)		0.559	0.03

^a^Model-estimated statistics are based on mixed-effects linear regression models examining change in CAPS-IV scores (past week) over time (baseline, after sessions 4 and 6, and posttreatment) with random intercepts and unstructured covariance structure. Intent to treat analysis: Exposure therapy (VRE vs. PE) by MDD interaction *p* value = 0.045; augmentation (DCS vs. placebo) by MDD interaction *p* value = 0.073. ES- standardized effect size (model-estimated between-group differences divided by the common standard deviation of the CAPS-IV changes scores baseline-posttreatment).

#### Augmentation (DCS vs. placebo)

There was a significant effect of time (F = 50.54, *p* < 0.001), but no significant effect of augmentation (F = 0.08, *p* = 0.774) nor augmentation-by-time interaction (F = 0.65, *p* = 0.422). Symptom improvements were 18.88 points in DCS and 22.14 points in placebo (model-estimated CAPS mean difference at posttreatment M = 3.80 [95% CI 0.03 to 7.57]. There was a significant main effect of baseline MDD (F = 25.50, *p* < 0.001). Augmentation-by-MDD interaction (F = 3.24, *p* = 0.073) suggested that depressed participants improved more on placebo (CAPS mean difference at posttreatment M = −8.43 [95% CI −10.98 to −5.88], *p* < 0.001, ES = −0.42), but DCS and placebo were equally effective for nondepressed participants (CAPS mean difference at posttreatment M = 0.75 [95% CI −1.81 to 3.30], *p* = 0.559, ES = 0.03).

#### Augmentation and mode of exposure therapy combined

The final sample size did not allow for inferences on a three-way interaction between MDD, exposure type, and augmentation, and the consistent MDD effects necessitated its inclusion in all models. Therefore, we present descriptive statistics only (Table S5; Fig. S3).

### Completer analyses

Secondary analysis of the treatment completers had a nearly identical pattern of results (Appendix S1).

### Secondary outcomes

Secondary analyses of self-reported post-traumatic stress and depressive symptoms were similar to the primary outcome (Fig. S2, Tables S3, S4).

### Genetic analyses

**Fig. 3:**
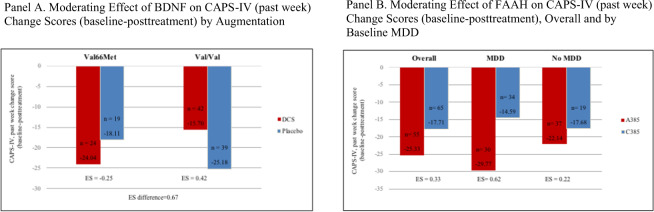
Genetic markers as moderators of treatment response. *Note*: Genetic markers (**A** Val66Met and **B** C385A) as moderators of treatment response. BDNF Val66Met (*n* = 59) is Val/Met (*n* = 50) and Met/Met (*n* = 9) carriers combined, FAAH C385A (*n* = 80) is A/A (*n* = 15) and A/C carriers (*n* = 65) combined. d = effect sizes (Cohen’s d) for CAPS-IV, past week baseline-posttreatment change scores for those with and without the genetic marker. CAPS-IV clinician administered posttraumatic stress disorder (PTSD) scale for DSM-IV, DCS D-cycloserine, MDD major depressive disorder.

For exploratory genotypic analyses, due to the small number of homozygotes for both BDNF and FAAH polymorphisms, variant genotype carriers were combined in all analyses of human data (Fig. 3). As expected, the BDNF Met66 allele (Val/Met and Met/Met combined) was present in 32.1% (*n* = 59) of the overall sample, equally distributed between augmentation groups (DCS 49.6%, placebo 50.4%, *p* = 0.786). Met66 was not associated with baseline CAPS scores (Met66 M = 72.34, SD = 19.26; Val/Val M = 73.66, SD = 19.63, *p* = 0.670), or MDD (Met66 *n* = 68, 54.4%; Val/Val *n* = 32, 54.2%, *p* = 0.984). Participants possessing one or more Met66 alleles improved more on DCS (ES = −0.25), while Val/Val carriers improved more on placebo (ES = 0.42). The apparent moderating effect of Val66Met on augmentation was substantial, with an effect size difference of 0.67 (Fig. 3).

As expected, the FAAH A385 allele was present in 44.9% (*n* = 80) of the sample. It was not associated with baseline CAPS (A385 M = 73.21, SD = 20.09, C385 M = 73.12, SD = 19.33) or MDD (A385 *n* = 44, 55%, C385 *n* = 54, *p* = 0.989). Across treatment groups, FAAH A385 allele carriers improved more compared to the C385 group (ES = 0.33), especially among the depressed group (ES = 0.62) (Fig. 3).

## Discussion

These results advance differential therapeutics for PTSD by showing that participants fared differently in each treatment according to comorbid depression status and genotype. Depressed participants improved more in VRE (ES = 0.14), while nondepressed participants improved more in PE (ES = −0.44). This suggests that comorbid MDD may be a critical variable in treatment selection.

Although sample size and possible population substructure in our sample limit our conclusions, the genetic analysis further supports differential therapeutics and underscores comorbid MDD as a treatment selection factor. Consistent with recent findings, showing that FAAH variants are associated with fear acquisition and extinction learning [27, 28] in experimental and human models, the FAAH A385 carriers improved more compared to non-carriers, particularly among participants who had MDD.

The moderating effect of BDNF on augmentation suggests that DCS may rescue the deficit in extinction learning for the BDNF Met66 carriers. These findings suggest that common genetic variants may play a role in PTSD treatment response. Of note, both human variants have already been validated to alter fear responses in multiple human studies [16, 29, 30], as well as alter brain levels of the protein [31].

This is the largest randomized controlled trial to date to compare VRE and PE, and the results equally support both types of exposure therapy as efficacious treatments for combat-related PTSD. Given the high rates of comorbidity of MDD in PTSD populations, we stratified our sample according to MDD a priori, and found that depressed participants improved more in VRE than in PE. VR has not been used to treat MDD in clinical trials. We speculate that the activity required to navigate the virtual environment may have facilitated behavioral activation in patients who received VRE. The novelty of using VR and being immersed in a virtual environment may be especially valuable or engaging to depressed patients, who exhibit altered reward processing and may therefore require greater stimulation to overcome negative experiences [32]. We speculate that VRE may more successfully engage depressed patients in treatment, given their relatively low dropout rate. This warrants further study.

Our results comparing DCS to placebo were not significant. A recent meta-analysis [12] found that the DCS augmentation effect was robust only if ingested at least 60 minutes prior to exposure, suggesting that our timing of DCS was not optimal. For example, Difede et al. [10] patients ingested DCS 90 minutes prior to exposure, and patients in the DCS VRE group showed significantly greater reduction in PTSD symptoms compared to the placebo control VRE group. Moreover, the consistent differential effect of comorbid depression on treatment outcome suggests that our planned secondary analysis assessing whether DCS is differentially beneficial in VRE vs. PE would have been substantially confounded. Depressed participants improved more in VRE vs. PE, and more in Placebo vs. DCS; that pattern was nearly reversed in nondepressed participants. To appropriately explore the joint effects of DCS and mode of exposure would have necessitated including MDD as an additional between-subjects factor in a three-way interaction, which we were not adequately powered to do. Future studies should examine these questions.

The original power analysis laid out the sample size necessary to detect the smallest clinically meaningful effect, but of course does not preclude the detection of larger effects. Examination of the study results suggests the following: 1) the pre-post treatment effect size within both VRE and PE was sufficiently large such that the smaller sample size did not diminish our ability to detect statistical significance, and 2) the effect sizes between VRE and PE, and between DCS and Placebo, were small enough (that is, the treatments were equally and highly effective) such that achieving the original target sample size would not have made a difference regarding detection of statistical significance. We did, in fact, examine the size of the effect we would have been able to detect keeping the original power analysis parameters the same but changing the sample size to the final study N of 132 Completers. The study was sufficiently powered to detect an effect of 0.46, that is, a change of just under half a standard deviation on the CAPS, or a medium effect. Many RCTs are powered on this sized effect, increasing our confidence in the validity of the findings.

Our study shows the utility of DCS for a defined subset of PTSD participants. It also supports the efficacy of both VRE and PE treatments. Furthermore, our study provides descriptive data for differential treatment effects in DCS-augmented VRE and PE for PTSD with MDD. The low dropout rate in VRE-DCS is consistent with our prior study of WTC survivors where there were no dropouts in the VRE-DCS condition [10].

Though participants preferred VRE, treatment response did not differ between VRE and PE. Nonetheless there is compelling evidence that patients are motivated to participate in treatments that they select [33] and that matching patients to preferred treatments has a positive effect on outcomes [34]. Offering patients their preferred treatment may be especially effective at motivating them to attend evidence-based treatments. Consistent with our prior study [10] there was a substantially lower dropout rate in the VRE + DCS group, suggesting some synergistic benefit of this combination. If VRE increases completion rates, that would be valuable, because currently a large percentage of exposure therapy patients do not complete treatment, and completers presumably have better outcomes than dropouts.

The study population was primarily male military service members who served recently; this may limit generalizability. Finally, while treatment was planned to be completed within 9 weeks, participants completed the 9 sessions on average over 16 weeks (SD = 9.28), although time in treatment did not differ between conditions.

Finally, a word about the limitation of our exploratory genetic results. At the time this project was funded, the candidate gene approach was dominant, and Genome Wide Association Studies (GWAS) methodology had not yet enjoyed widespread feasibility or adoption. During the seven years of data collection for this study, the prevailing view on genetic assay methodology and technology changed significantly [35] and the candidate gene approach had fallen out of favor as more robust and precise methodologies became available and were widely adopted. Nonetheless, the candidate gene approach used in this study had been peer-reviewed during the grant review process and the data generated are not post-hoc, but are based on a priori exploratory hypotheses, which were generated from findings [36] using a combination of normative human and pre-clinical models. During the life of this study, several human and pre-clinical studies have been published, which have provided convergent evidence, which are consistent with our data [27, 28, 37]. We argue that while there are limitations of the single candidate gene approach, our data is consistent with convergent evidence in subsequently published studies, and it was generated based on a priori hypotheses. We suggest that our results should be given due consideration in the context of our overall study design and findings. The likelihood of our results being spurious is meaningfully reduced by the convergent evidence yielded from these more recent studies [27, 28, 37].

These results support the promise of differential therapeutics for PTSD. This study provides strong evidence that MDD status should be considered in treatment selection for exposure therapies for PTSD as it affected both DCS augmentation outcomes and psychotherapeutic outcomes. The exploratory genetic analyses also provide nascent support for differential therapeutics. Both candidate genes (BDNF Val66Met and FAAH C385A) warrant further research in clinical trials for PTSD. Finally, future studies should consider genomic profiles when including the use of DCS as a cognitive enhancer.

## Supplementary information

Supplementary Online Content
