# Supplementary material for: Enhancing exposure therapy for posttraumatic stress disorder (PTSD): a randomized clinical trial of virtual reality and imaginal exposure with a cognitive enhancer
Source: Transl Psychiatry. 2022 Jul 27;12:299. doi: 10.1038/s41398-022-02066-x (PMC9329292; doi:10.1038/s41398-022-02066-x)
Supplement: Supplementary file 1 — Supplementary Online Content [file 41398_2022_2066_MOESM1_ESM.pdf]

Supplementary Online Content

Figure S1. Treatment Preference, Overall and by Baseline MDD

Table S1. Treatment Preference by Baseline Demographic, Military Service and Clinical Characteristics

Table S2. Dropout by Baseline Demographic, Military Service and Clinical Characteristics

Appendix S1. Completer Analyses

Figure S2. Cross-sectional Mean PCL and BDI scores: Exposure Therapy and Augmentation Over Time, by Baseline MDD

Table S3. Descriptive and Model-estimated Statistics for Secondary Outcomes (PCL): Exposure Therapy and Augmentation Over Time by Baseline MDD

Table S4. Descriptive and Model-estimated Statistics for Secondary Outcomes (BDI): Exposure Therapy and Augmentation over Time by Baseline MDD

Table S5. Descriptive Statistics for Primary Outcome (CAPS-IV, past week) by Exposure Therapy and Augmentation (combined) Over Time, Overall and by Baseline MDD

Figure S3. Descriptive Statistics for Primary Outcome (CAPS-IV, past week) by Exposure Therapy and Augmentation (combined) Over Time, Overall and by Baseline MDD

Figure S4. Cross-sectional Mean End-of-session SUDS (Subjective Units of Distress scale) by Augmentation Over Time, Overall and by Baseline MDD

Appendix S2. SUDS Analyses (DCS vs. Placebo)

Figure S1. Treatment Preference, Overall and by Baseline MDD

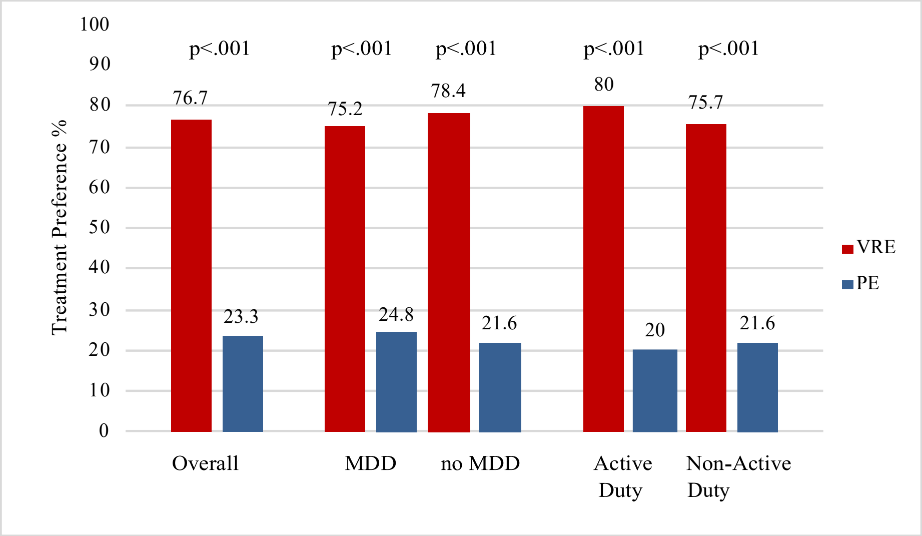

Note. P-values are based on chi-square goodness of fit tests. Overall: preferred VRE, received VRE-51%; preferred PE, received PE-50%, χ^2 =0.014, p=.904. Abbreviations: VRE-virtual reality exposure therapy; PE-prolonged imaginal exposure therapy; MDD- major depressive disorder.

Table S1. Treatment Preference by Baseline Demographic, Military Service and Clinical Characteristics

	Preferred ,VRE n=145 (75.5%)		Preferred PE n=44 (22.9%)		p-value
**Demographic characteristics**					
Age M (SD)	34.28	7.55	35.41	8.52	.403
Male n (%)	132	91.0	38	86.4	.367
Ethnicity/Race n (%)					.707
White	69	47.6	17	38.6	
African American/Black	21	14.5	7	15.9	
Hispanic/Latino	38	26.2	15	34.2	
Other	17	11.7	5	11.4	
Education n (%)					.787
High school or GED	21	14.5	8	18.2	
Some college/training	81	55.9	22	50.0	
College graduate	26	17.9	7	15.9	
More than college	17	11.7	7	15.9	
Relationship status n (%)					.280
Single	43	29.7	14	31.8	
Married/Live w/significant other	74	51.0	26	59.1	
Separated/Divorced/Widowed	28	19.3	4	9.1	
**Military characteristics**					
Military service n (%)					.588
OEF only	31	21.4	8	18.2	
OIF only	63	43.4	23	52.3	
Both OEF and OIF	51	35.2	13	29.5	
Branch of the Armed Forces n (%)					.138
Army	81	55.9	30	68.2	
Marines	43	29.7	12	27.3	
Navy	15	10.3	0	0	
Air Force	6	4.1	2	45.0	
Other	1	100	0	0	
Number of deployments n (%)					.920
1	51	35.2	17	38.6	
2	50	34.5	16	36.4	
3	21	14.5	5	11.4	
4 or more	23	15.9	6	13.6	
Months in theater M (SD)	19.02	12.45	19.55	16.50	.821
Active duty n (%)	36	24.8	9	20.5	.551
**Clinical characteristics**					
Past PTSD treatment n (%)	90	62.1	25	56.8	.532
Baseline MDD n (%)	76	52.4	25	56.8	.608
CAPS-IV, past week M(SD)	72.77	19.08	73.00	20.36	.946
CAPS-IV, past week, at posttreatment (model-estimated)	51.02	8.75	53.83	7.64	.102

Note. P-values are based on independent samples t-tests or chi-squared tests. Abbreviations: VRE-virtual reality exposure therapy; PE-prolonged imaginal exposure therapy; MDD-major depressive disorder; CAPS-IV- Clinician Administered Posttraumatic Stress Disorder (PTSD) Scale for DSM-IV; OEF and OIF - Operations Iraqi Freedom and Enduring Freedom.

Table S2. Dropout by Baseline Demographic, Military Service and Clinical Characteristics

	Completer n=132 (68.8%)		Dropout n=60 (31.3%)		p-value
**Treatment group**					
VRE n (%)	70	72.2	27	27.8	.302
PE n (%)	62	65.3	33	34.7	
DCS n (%)	70	73.7	25	26.3	.144
Placebo n (%)	62	63.9	35	36.1	
VRE+DCS n (%)	39	83.0	8	17.0	0.109
VRE+Placebo n (%)	31	62.0	19	38.0	
PE+DCS n (%)	31	64.6	17	35.4	
PE+Placebo n (%)	31	66.0	16	34.0	
**Clinical characteristics**					
Baseline MDD n (%)	70	67.3	34	32.7	.639
No Baseline MDD n (%)	62	70.5	26	29.5	
Baseline MDD					
VRE n (%)	36	66.7	17	33.3	.885
PE n (%)	34	68.0	16	32.0	
DCS n (%)	38	73.1	14	26.9	.210
Placebo n (%)	32	61.5	20	38.5	
No Baseline MDD					
VRE n (%)	34	79.1	9	20.9	.083
PE n (%)	28	62.2	17	37.8	
DCS n (%)	32	74.4	11	25.6	.426
Placebo n (%)	30	66.7	15	33.3	
CAPS-IV, past week M(SD)	72.82	19.59	73.52	19.35	.818
Physical or sexual trauma n (%)	77	62.6	46	37.4	.019
No physical or sexual trauma n (%)	53	79.1	14	20.9	
**Demographic characteristics**					
Age M (SD)	35.09	8.18	33.56	6.80	.181
Male n (%)	119	69.2	53	30.8	.702
Female n (%)	13	65.0	7	35.0	
Ethnicity/Race n (%)					.097
White	67	76.1	21	23.9	
African American/Black	21	72.4	8	27.6	
Hispanic/Latino	30	56.6	23	43.4	
Other	14	63.6	8	36.4	
Education n (%)					.097
High school or GED	18	62.1	11	37.9	
Some college/training	66	63.5	38	36.5	
College graduate	27	81.8	6	18.2	
More than college	21	80.8	5	19.2	
Relationship status n (%)					.478
Single	40	67.8	19	32.2	
Married/Live with significant other	67	67.0	33	33.0	
Separated/Divorced/Widowed	25	78.1	7	21.9	
**Military characteristics**					
Military Service n (%)					.074
OEF only	24	61.5	15	38.5	
OIF only	55	64.0	31	36.0	
Both OEF and OIF	53	79.1	14	20.9	
Number of deployments n (%)					.961
1	47	69.1	21	30.9	
2	45	68.2	21	31.8	
3	17	65.4	9	34.6	
4 or more	23	71.9	9	28.1	
Months in Theater M (SD)	19.45	13.80	18.62	12.81	.683
Active duty n (%)	36	78.3	10	21.7	.111
Non-Active duty n (%)	96	65.8	50	34.2	
**Treatment preference and Treatment received, Treatment satisfaction**					
Preferred VRE n (%)	99	68.3	46	31.7	.785
Preferred PE n (%)	31	70.5	13	29.5	
Preferred VRE, received VRE n (%)	53	71.6	21	28.4	.612
Preferred PE, received PE n (%)	14	63.6	8	36.4	
Preferred VRE, received PE n (%)	46	64.8	25	35.2	
Preferred PE, received VRE n (%)	31	70.5	13	29.5	
Treatment satisfaction M(SD)	28.58	3.33	27.23	5.34	.387

Note: P-values are based on independent samples t-tests or chi-squared tests. Treatment satisfaction for dropouts is based on the last satisfaction rating available. Abbreviations: VRE-virtual reality exposure therapy; PE-prolonged imaginal exposure therapy; DCS-D- Cycloserine; MDD-major depressive disorder; SSRI- Selective serotonin reuptake inhibitor; CAPS-IV- Clinician Administered Posttraumatic Stress Disorder (PTSD) Scale for DSM-IV; OEF and OIF - Operations Iraqi Freedom and Enduring Freedom.

Appendix S1. Completer Analyses. Identical to ITT findings, completer analyses suggested PE was more effective for non-depressed participants (model estimated mean CAPS difference at posttreatment for completers M=12.01, p<.001, ES=-.60) but VRE was more effective for depressed subjects (model estimated mean CAPS difference at posttreatment M=7.65, p<.001, ES=.31). Furthermore, there was a significant effect of time (F=44.09, p<.001), a non-significant effect for augmentation (F=.132, p=.716) and a non- significant augmentation by time interaction (F=.422, p=.517). There was a significant main effect of baseline MDD (F=19.60, p<.001) and a marginally significant augmentation by MDD interaction (F=2.97, p=.087) suggesting that depressed participants improved more on placebo (model estimated mean CAPS difference at posttreatment M=10.55, p<.001, ES=-.53. However, DCS was slightly more effective for completer participants who were non-depressed (model estimated mean CAPS difference at posttreatment M=4.26, p=.035, ES=.17).

Figure S2. Cross-sectional Mean PCL and BDI scores: Exposure Therapy and Augmentation Over Time, by Baseline MDD

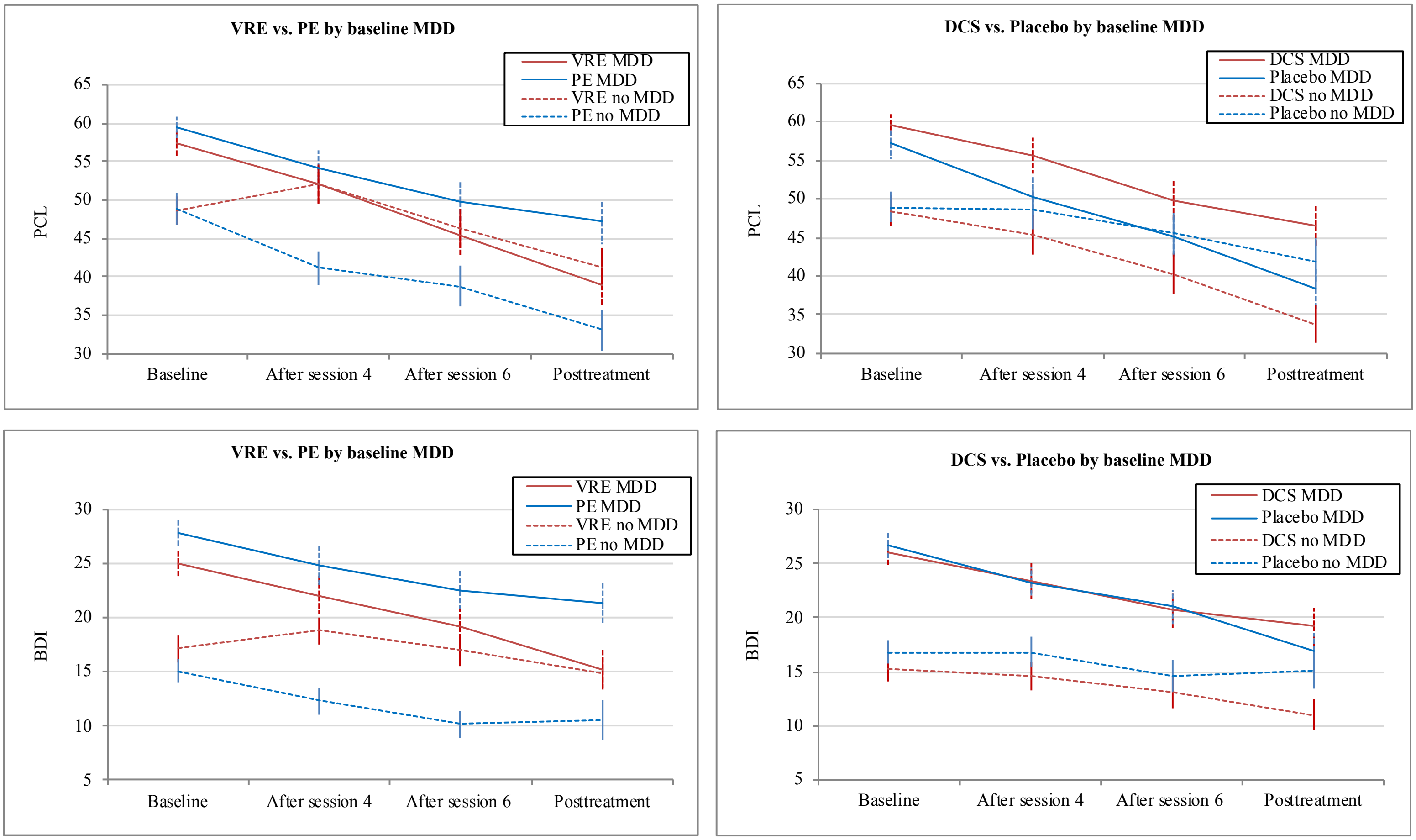

Note. Bars represent standard errors. Abbreviations: VRE-virtual reality exposure therapy; PE-prolonged imaginal exposure therapy; DCS- D-Cycloserine; MDD-major depressive disorder.

Table S3. Descriptive and Model-estimated Statistics for Secondary Outcomes (PCL): Exposure Therapy and Augmentation Over Time by Baseline MDD

	Exposure Therapy (VRE vs. PE)						Augmentation (DCS vs. Placebo)					
	VRE n=97			PE n=95			DCS n=95			Placebo n=97		
**PCL**	**Descriptive Statistics**						**Descriptive Statistics**					
**Overall**	**N**	**M**	**SD**	**N**	**M**	**SD**	**N**	**M**	**SD**	**N**	**M**	**SD**
Baseline	95	53.35	12.56	93	54.43	12.89	93	54.51	12.70	95	53.27	12.75
After session 4	78	52.03	14.96	69	48.25	15.03	74	51.03	15.24	73	49.47	14.95
After session 6	72	45.82	15.92	64	44.92	15.83	69	45.51	16.45	67	45.28	15.28
Posttreatment	67	40.01	14.94	56	41.16	16.41	66	40.91	16.04	57	40.11	15.128
3-month Follow-up	61	42.38	15.97	50	42.64	17.72	59	43.36	17.32	52	41.52	16.39
**MDD**												
Baseline	52	57.33	11.50	49	59.45	9.90	51	59.53	10.13	50	57.16	11.33
After session 4	43	51.95	15.36	38	54.03	14.86	41	55.56	14.49	40	50.23	15.35
After session 6	39	45.38	17.01	36	49.72	15.72	38	49.74	16.81	37	45.14	15.93
Posttreatment	34	38.85	15.48	32	47.19	16.26	37	46.49	16.26	29	38.31	15.41
3-month Follow-up	31	42.06	17.92	27	48.85	19.19	31	48.97	18.14	27	40.93	18.67
**No MDD**												
Baseline	43	48.53	12.22	44	48.84	13.62	42	48.40	12.93	45	48.96	12.97
After session 4	35	52.11	14.69	31	41.16	12.07	33	45.39	14.44	33	48.55	14.62
After session 6	33	46.33	14.77	28	38.75	13.94	31	40.32	14.63	30	45.47	14.71
Posttreatment	33	41.21	14.51	24	33.13	13.05	29	33.79	12.82	28	41.96	14.92
3-month Follow-up	30	42.70	13.99	23	35.35	12.70	28	37.14	14.16	25	42.16	13.20

**PCL**	**Model-estimated statistics[a] at posttreatment**				**Model-estimated statistics[a] at posttreatment**			
	Mean difference (VRE vs. PE)	95%CI	p-value	ES	Mean difference (DCS vs. Placebo)	95%CI	p-value	ES
MDD								
Intent-to-treat analysis	3.98	(1.68, 6.28)	0.001	0.30	-4.40	(-6.78, -2.02)	<.001	-0.33
No MDD								
Intent-to-treat analysis	-2.71	(-5.04, -.39)	0.023	-0.21	3.57	(1.20, 5.94)	0.004	0.28

Note. [a]Model-estimated statistics are based on mixed effects linear regression models examining change in PCL scores over time (baseline, after sessions 4 and 6, and posttreatment) with random intercepts and unstructured covariance structure. Intent to treat analysis PCL: Exposure therapy (VRE vs. PE) by MDD interaction p-value =.091; augmentation (DCS vs. placebo) by MDD interaction p-value =.021. ES- standardized effect size (model-estimated between-group differences divided by the common standard deviation of the PCL changes scores baseline-posttreatment). Abbreviations: VRE-virtual reality exposure therapy; PE-prolonged imaginal exposure therapy; DCS- D-Cycloserine; MDD-major depressive disorder.

Table S4. Descriptive and Model-estimated Statistics for Secondary Outcomes (BDI): Exposure Therapy and Augmentation over Time by Baseline MDD

	Exposure Therapy (VRE vs. PE)						Augmentation (DCS vs. Placebo)					
	VRE n=97			PE n=95			DCS n=95			Placebo n=97		
**BDI**	Descriptive Statistics						Descriptive Statistics					
	N	M	SD	N	M	SD	N	M	SD	N	M	SD
Baseline	94	21.49	9.26	93	21.81	10.24	93	21.22	9.96	94	22.07	9.54
After session 4	78	20.56	9.85	69	19.17	11.35	74	19.47	10.41	73	20.36	10.78
After session 6	72	18.19	10.34	64	17.14	10.79	69	17.26	9.90	67	18.15	11.19
Posttreatment	67	15.03	9.37	56	16.70	11.63	66	15.59	10.36	57	16.02	10.63
3-month Follow-up	61	15.92	10.93	50	16.16	12.37	59	16.31	11.35	52	15.71	11.88
**MDD**												
Baseline	52	25.02	8.56	49	27.84	8.52	51	26.08	8.43	50	26.70	8.87
After session 4	43	22.02	11.36	38	24.79	11.23	41	23.37	10.81	40	23.28	11.95
After session 6	39	19.23	11.63	36	22.56	10.36	38	20.66	9.78	37	21.00	12.43
Posttreatment	34	15.18	10.52	32	21.34	11.27	37	19.19	10.78	29	16.86	11.87
3-month Follow-up	31	17.45	13.09	27	20.93	13.23	31	20.65	11.97	27	17.26	14.43
**No MDD**												
Baseline	42	17.12	8.24	44	15.09	7.48	42	15.31	8.40	44	16.82	7.37
After session 4	35	18.77	7.36	31	12.29	6.91	33	14.64	7.58	33	16.82	8.00
After session 6	33	16.97	8.57	28	10.18	6.56	31	13.10	8.47	30	14.63	8.36
Posttreatment	33	14.88	8.17	24	10.50	9.06	29	11.00	7.80	28	15.14	9.32
3-month Follow-up	30	14.33	8.05	23	10.57	8.55	28	11.50	8.48	25	14.04	8.28

**BDI**	Model-estimated statistics[a] at posttreatment				Model-estimated statistics[a] at posttreatment			
	Mean difference (VRE vs. PE)	95%CI	p-value	ES	Mean difference (DCS vs. Placebo)	95%CI	p-value	ES
MDD								
Intent-to-treat analysis	4.20	(2.79, 5.62)	<.001	0.46	0.60	(-.93, 2.13)	0.435	0.07
No MDD								
Intent-to-treat analysis	-2.56	(-4.05, -1.08)	0.001	-0.36	3.43	(1.89, 4.97)	<.001	0.49

Note. [a]Model-estimated statistics are based on mixed effects linear regression models examining change in BDI scores over time (baseline, after sessions 4 and 6, and posttreatment) with random intercepts and unstructured covariance structure. Intent to treat analysis BDI: Exposure therapy (VRE vs. PE) by MDD interaction p-value =.012; augmentation (DCS vs. placebo) by MDD interaction p-value =.241. ES- standardized effect size (model-estimated between-group differences divided by the common standard deviation of the BDI changes scores baseline-posttreatment).

Table S5. Descriptive Statistics for Primary Outcome (CAPS-IV, past week) by Exposure Therapy and Augmentation (combined) Over Time, Overall and by Baseline MDD

	PE+Placebo n=47			PE+DCS n=48			VRE+Placebo n=50			VRE+DCS n=47		
**CAPS-IV, past week**												
**Overall**	**N**	**M**	**SD**	**N**	**M**	**SD**	**N**	**M**	**SD**	**N**	**M**	**SD**
Baseline	47	70.74	20.37	48	75.08	18.69	50	74.46	19.11	47	71.72	19.98
After session 4	36	66.39	24.54	32	77.56	23.40	40	77.08	20.04	41	76.63	19.98
After session 6	33	61.39	26.98	31	68.42	26.52	34	62.71	23.28	39	63.21	24.41
Posttreatment	31	44.16	23.15	30	57.27	25.90	30	53.17	25.82	39	51.85	27.43
3-month Follow-up	26	40.46	25.64	26	60.23	29.11	28	50.64	27.26	36	47.78	25.51
MDD												
Baseline	25	78.16	18.53	25	85.64	15.39	27	79.15	17.15	27	77.74	17.55
After session 4	19	74.26	24.70	19	88.84	15.98	23	77.00	21.80	22	79.68	23.11
After session 6	18	68.39	24.21	18	78.67	22.84	19	59.89	24.61	20	66.25	26.79
Posttreatment	16	53.50	23.40	18	69.11	22.66	15	47.67	27.21	20	57.00	30.38
3-month Follow-up	13	45.46	30.65	15	72.93	24.35	14	50.21	29.36	17	54.06	30.00
No MDD												
Baseline	22	62.32	19.42	23	63.61	14.97	23	68.96	20.18	20	63.60	20.59
After session 4	17	57.59	21.79	13	61.08	23.14	17	77.18	18.03	19	73.11	15.47
After session 6	15	53.00	28.52	13	54.23	25.37	15	66.27	21.79	19	60.00	21.88
Posttreatment	15	34.20	18.87	12	39.50	20.03	15	58.67	24.00	19	46.42	23.53
3-month Follow-up	13	35.46	19.39	11	42.91	26.76	14	51.07	26.11	19	42.16	19.85

Note: Abbreviations: VRE-virtual reality exposure therapy; PE-prolonged imaginal exposure therapy; DCS-D-Cycloserine; CAPS-IV- Clinician Administered Posttraumatic Stress Disorder (PTSD) Scale for DSM-IV; MDD-major depressive disorder.

Figure S3. Descriptive Statistics for Primary Outcome (CAPS-IV, past week) by Exposure Therapy and Augmentation (combined) Over Time, Overall and by Baseline MDD

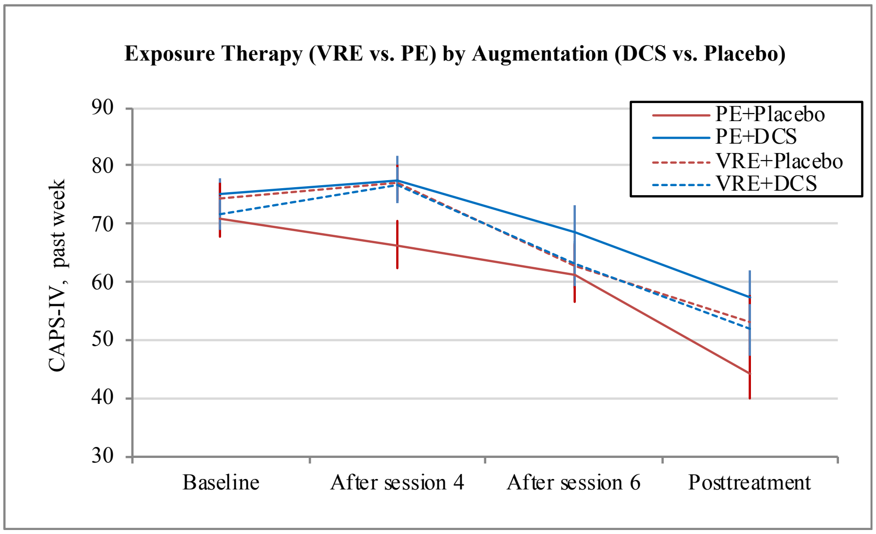

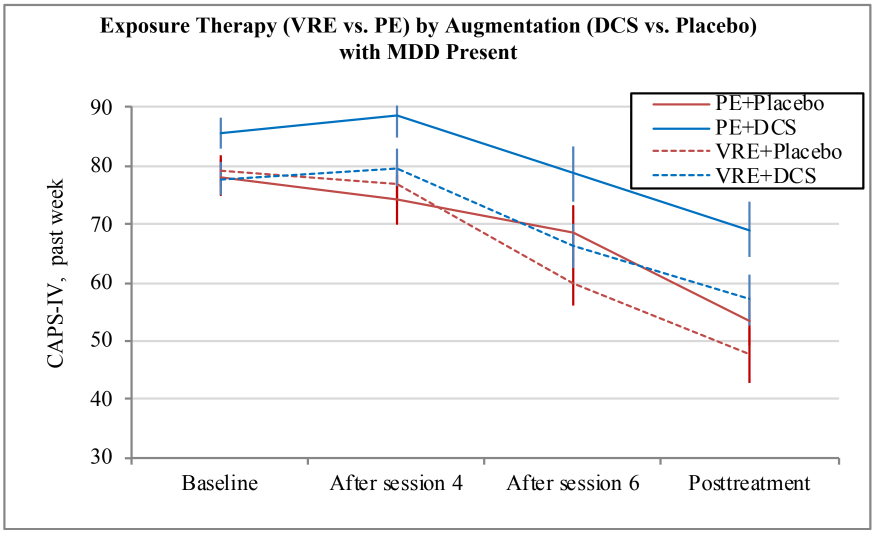

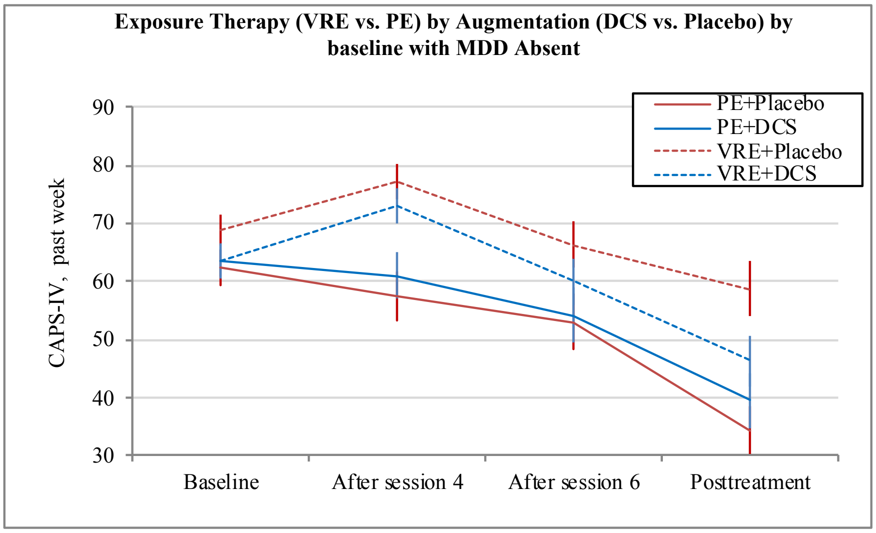

Note: Abbreviations: VRE-virtual reality exposure therapy; PE-prolonged imaginal exposure therapy; DCS-D-Cycloserine; CAPS-IV- Clinician Administered Posttraumatic Stress Disorder (PTSD) Scale for DSM-IV; MDD-major depressive disorder.

Figure S4. Cross-sectional Mean End-of-session SUDS (Subjective Units of Distress scale) by Augmentation Over Time, by Baseline MDD

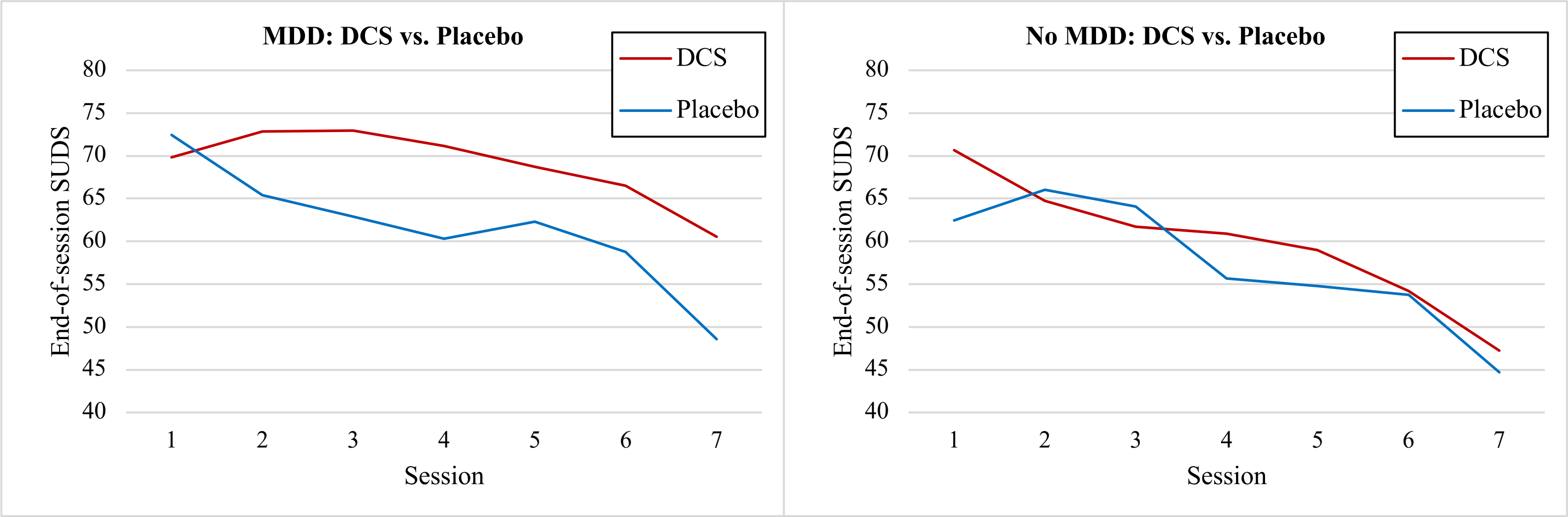

Note: Abbreviations: SUDS-(Subjective Units of Distress Scale, 0=no anxiety and 100=maximum anxiety); DCS- D-Cycloserine; MDD-major depressive disorder.

Appendix S2. SUDS Analyses (DCS vs. Placebo). We focused on the analysis of the between session extinction (as measure by end-of-session SUDS), since recent literature suggests that between session extinction is the primary mechanisms underlying exposure therapy for the treatment of PTSD (Rauch et al., 2018).

Multilevel modeling (SUDS nested within person over time) was used to evaluate patterns of between-session extinction in the DCS and placebo groups overall and by MDD status. Models included fixed effects of time (session number), group (DCS vs. placebo) and time by group intervention. Baseline MDD status was included as a fixed effect. Models were then stratified by MDD status, following a significant MDD effect. Next CAPS change from baseline to posttreatment was added as a fixed effect.

Findings indicate that end-of-session SUDS decreased over time (session effect p<. 001, average decrease was 18.11 points). There was no significant effect of the medication (DCS vs. placebo) on the reduction in end-of-session SUDS over the course of the treatment (interaction p-value >.05, reduction from session 3 to session 9 in DCS group was 15.02 points, reduction in placebo group was 21.22 points). Among participants without MDD (MDD-), end-of-session SUDS decrease over time was similar between DCS and placebo groups (22.97 points and 17.96, respectively). Among participants with MDD (MDD+), end-of-session SUDS decrease over time was 23.31 points in the placebo group, compared to only 8.66 points in DCS group (Figures S4). Overall, symptoms reduction (CAPS change from baseline to posttreatment) was associated with a decrease in between-session habituation (p=.007). However, this was evident among participants without depression (p=.007) but not among participants with depression (p=.206).
